# How Genes Meet Diet in LCHAD Deficiency: Nutrigenomics of Fatty Acid Oxidation Disorder

**DOI:** 10.3390/ijms262010140

**Published:** 2025-10-18

**Authors:** Zdzislaw Kochan, Joanna Karbowska

**Affiliations:** 1 Laboratory of Nutritional Biochemistry, Department of Clinical Nutrition, Medical University of Gdansk, 80-211 Gdansk, Poland; 2 Department of Biochemistry, Medical University of Gdansk, 80-211 Gdansk, Poland

**Keywords:** mitochondrial diseases, fatty acid oxidation, medium-chain fatty acids, MCT, nutritional therapy, nutritional regulation, human

## Abstract

Mitochondrial long-chain fatty acid β-oxidation supplies energy to the heart, liver, and skeletal muscle. Impairment of this process due to a block at the step catalyzed by long-chain 3-hydroxyacyl-CoA dehydrogenase (LCHAD) leads to bioenergetic failure, manifesting as hypoglycemia, recurrent rhabdomyolysis, cardiomyopathy, and hepatic dysfunction. Accumulation of toxic intermediates—long-chain 3-hydroxyacyl-CoAs and the corresponding 3-hydroxyacylcarnitines—contributes to pigmentary retinopathy and peripheral neuropathy. Early diagnosis and careful dietary management can reduce life-threatening decompensation in childhood and improve survival into adulthood. This review examines the genetics of human LCHAD deficiency, describes its multisystem complications, and outlines nutritional strategies used to bypass the enzymatic block. We also explore nutrigenomic signals elicited by dietary treatment in LCHAD deficiency.

## 1. Introduction

Long-chain 3-hydroxyacyl-CoA dehydrogenase (LCHAD) is a component of the mitochondrial trifunctional protein (MTP) that catalyzes the last three of the four consecutive reactions in mitochondrial β-oxidation of long-chain fatty acids [1]. The oxidative catabolism of long-chain fatty acids in mitochondria supplies much of the energy needed by the heart, the liver, and the skeletal muscle; disruption or reduced efficiency of this pathway can therefore impair the bioenergetics of these tissues, leading to cardiac, hepatic, and muscular dysfunction. Consistently, among its diverse clinical features, LCHAD deficiency most often presents with heart muscle disease, liver failure, hypoketotic hypoglycemia, and repeated episodes of muscle fiber breakdown [2]. Furthermore, the accumulation of toxic intermediates produced by the defective β-oxidation pathway—primarily long-chain 3-hydroxyacyl-CoAs and the corresponding 3-hydroxyacylcarnitines—can lead to peripheral neuropathy and pigmentary retinopathy. Early detection of the disease and meticulous dietary management can prevent fatal metabolic decompensation in children and enable them to reach adulthood.

## 2. Human Mitochondrial β-Oxidation Systems

Two β-oxidation systems operate in human mitochondria (Table 1). The first consists of enzymes anchored to the inner mitochondrial membrane and primarily oxidizes very-long- and long-chain fatty acids (VLCFAs and LCFAs) derived from the diet or synthesized endogenously. Of the four reactions in each β-oxidation cycle, the initial dehydrogenation step is catalyzed by very-long-chain acyl-CoA dehydrogenase (VLCAD) and long-chain acyl-CoA dehydrogenase (LCAD), which have partially overlapping substrate specificities [3]. ACAD9, a later-identified member of the human acyl-CoA dehydrogenase family, shares high sequence homology with VLCAD [4] but appears to function predominantly in mitochondrial complex I assembly rather than in long-chain acyl-CoA β-oxidation [5,6]. The remaining three reactions in this very-long- and long-chain β-oxidation system are carried out by enzymatic domains of MTP, encoded by *HADHA* and *HADHB* [7,8]. These reactions are catalyzed by the long-chain enoyl-CoA hydratase (LCEH), long-chain 3-hydroxyacyl-CoA dehydrogenase (LCHAD), and long-chain 3-ketoacyl-CoA thiolase (LCKAT) activities. In addition, MTP’s monolysocardiolipin acyltransferase (MLCLAT) activity participates in cardiolipin remodeling [9].

After several cycles of long-chain β-oxidation, the resulting medium-chain acyl-CoA becomes the substrate for a second β-oxidation system comprising soluble, monofunctional enzymes in the mitochondrial matrix. In this system, the initial dehydrogenation is catalyzed by medium- or short-chain acyl-CoA dehydrogenase (MCAD or SCAD), depending on chain length.

All acyl-CoA dehydrogenases—VLCAD, ACAD9, LCAD, MCAD, and SCAD—transfer electrons to the respiratory chain via electron transfer flavoprotein (ETF) [15]. Among human β-oxidation enzymes, LCHAD is unique—no other enzyme oxidizes long-chain 3-hydroxyacyl-CoAs. Consequently, loss of this activity blocks mitochondrial β-oxidation of long-chain fatty acids.

## 3. The Genetic Architecture of LCHAD Deficiency

### 3.1. The HADHA Gene

Human LCHAD is the product of the *HADHA* gene located on chromosome 2 (2p23.3) [16]. *HADHA* is a highly polymorphic gene, with more than 280 reported pathogenic and likely pathogenic variants (Table 2) [17]. Pathogenic variants in *HADHA* inactivate the LCHAD catalytic site, creating an enzymatic block in mitochondrial β-oxidation of long-chain fatty acids. Consequently, long-chain 3-hydroxyacyl-CoAs accumulate in the matrix, deplete the intramitochondrial coenzyme A (CoA) pool, disrupt the inner membrane in a detergent-like manner, inhibit respiratory chain complexes, and ultimately impair mitochondrial function. The resulting energy deficit in hepatocytes, cardiomyocytes, and skeletal muscle fibers manifests clinically as hypoketotic hypoglycemia, cardiomyopathy, muscle weakness, and recurrent rhabdomyolysis. A portion of the accumulated 3-hydroxyacyl-CoAs is converted to the corresponding carnitine derivatives and exported to the cytosol and then into the bloodstream. These long-chain 3-hydroxyacylcarnitines can reach micromolar concentrations in plasma and add to systemic toxicity; they are arrhythmogenic and may contribute to neuroinflammation [18]. In addition, excess intracellular LCFAs and triglyceride overload trigger endoplasmic reticulum stress, promote hepatic steatosis and hepatomegaly, and further increase arrhythmia risk.

Because LCHAD deficiency is inherited in an autosomal-recessive manner, it manifests only in individuals with pathogenic *HADHA* variants on both alleles. Compound heterozygous *HADHA* variants can underlie isolated LCHAD deficiency; by contrast, compound heterozygous truncating or splice-disrupting variants in *HADHA* or *HADHB* more often cause complete MTP deficiency [19,20]. The canonical model involves biallelic pathogenic variants in a single gene (*HADHA* or *HADHB*); putative digenic mechanisms have been discussed in the mitochondrial genetics literature but are not established for MTP deficiency [21].

**Table 2:** Most frequent pathogenic variants in the *HADHA* gene associated with LCHAD deficiency.

Variant	Amino Acid Change	Consequence	Frequency	References
c.180+3A>G rs781222705	—	SNV, splice region	1:19,800	[22]
c.914T>A rs137852774	p.Ile305Asn (I305N)	SNV, missense	1:37,800	[23]
c.919-2A>G rs200017313	—	SNV, splice acceptor	1:24,900	[24]
c.1132C>T rs137852770	p.Gln378Ter (Q378*)	SNV, nonsense	1:66,200	[25]
c.1528G>C rs137852769	p.Glu510Gln (E510Q)	SNV, missense	1:720	[26]
c.1678C>T rs137852771	p.Arg560Ter (R560*)	SNV, nonsense	1:42,200	[27]
c.1793_1794del rs769580842	p.His598fs (H598fs)	Deletion, frameshift	1:24,881	[28]
c.1981_1999del rs749848370	p.Leu661fs (Y639fs)	Deletion, frameshift	1:40,400	[29]
c.2026C>T rs771028541	p.Arg676Cys (R676C)	SNV, missense	1:52,938	[30]
c.2225_2228dup rs868816467	p.Phe744fs (F744fs)	Duplication, frameshift	1:66,200	[31]

SNV, single-nucleotide variant. An asterisk (*) indicates a stop codon that terminates protein synthesis. Table created by the authors from the dbSNP database cited per row (see ‘References’ column).

### 3.2. Pathogenic Variant c.1528G>C

The pathogenic *HADHA* variant c.1528G>C (p.Glu510Gln) is the predominant cause of LCHAD deficiency in Europe but is rare or unobserved in East Asian cohorts [32,33]. This geographic split is consistent with a European founder mutation, likely originating around the Baltic Sea, that drifted to higher frequency locally, whereas East Asian populations lack this founder haplotype and show a different mix of *HADHA* and *HADHB* variants [34]. The highest documented carrier frequency (≈1:57) has been reported in the Kashubian ethnolinguistic community of northern Poland with historically limited gene flow [32,35]. In the broader Pomeranian province, the carrier frequency is ≈1:75 versus ≈1:187 elsewhere in Poland, supporting a regional founder effect (Table 3).

The c.1528G>C variant impairs the MTP α-subunit and is linked to defective cardiolipin remodeling with accumulation of immature, more saturated cardiolipin species, likely contributing to mitochondrial dysfunction in LCHAD deficiency [9,36].

**Table 3:** Carrier frequency of pathogenic variant c.1528G>C, p.Glu510Gln (E510Q), in European countries.

Country or Region	Carrier Frequency	References
Czechia	1:145	[37]
Denmark	1:172	[38]
Estonia	1:173	[39]
Finland, Northern	1:365	[40]
Finland, Western	1:132	[40]
Finland, Southern	1:164	[40]
Finland, Eastern	1:193	[40]
Germany	1:243	[41]
Poland, Kashubian	1:57	[32]
Poland, Pomeranian	1:75	[32]
Poland, rest of country	1:187	[32]
The Netherlands	1:680	[42]
Ukraine	1:288	[43]
United Kingdom	1:927	[17]
Worldwide	1:720	[17]

Table created by the authors from the studies cited per row (see ‘References’ column).

## 4. Multisystem Health Complications in LCHAD Deficiency

Newborn screening of dried blood spots typically detects elevated long-chain 3-hydroxyacylcarnitines—most notably C14-OH, C14:1-OH, C16-OH, C16:1-OH, C18-OH, and C18:1-OH—which prompt confirmatory testing [19,44,45,46]. Once LCHAD deficiency is diagnosed, earlier recognition and standardized dietary management are associated with improved survival [47,48,49]. Clinical manifestations of LCHAD deficiency include hypoglycemia, rhabdomyolysis, cardiomyopathy, hepatic dysfunction, retinopathy, and peripheral neuropathy [19,47,50,51,52,53]. Additional reported features are hypoparathyroidism, nephrotic syndrome, pancytopenia, and pulmonary involvement [19,47]. Clinical severity in LCHAD deficiency is heterogeneous; however, higher plasma concentrations of long-chain 3-hydroxyacylcarnitines correlate with worse retinal function and faster progression of pigmentary chorioretinopathy, and retinal involvement is most frequent in individuals homozygous for *HADHA* c.1528G>C [33,54]. In established patients, circulating lipid and acylcarnitine profiles reflect disease status and relate to measures of retinal function [47,50]. Acylcarnitines tend to rise during metabolic decompensation and decline with treatment [18,55].

In lipidomic studies, stable patients show lower mean total plasma lipid concentrations, likely reflecting adherence to low-fat diets [50]. There is also a well-described obstetric association—the fetal p.Glu510Gln (c.1528G>C) variant has been linked to maternal acute fatty liver of pregnancy (AFLP) and HELLP syndrome (hemolysis, elevated liver enzymes, low platelets) [56].

Cardiomyopathy in LCHAD deficiency can be life-threatening. In one family, after the death of a sibling with compound heterozygous *HADHA* variants, a second affected child was listed for heart transplantation and underwent the procedure at three years of age [57]. At age seven (four years post-transplant), neither cardiac nor extracardiac manifestations of LCHAD deficiency were reported [57]. Although generalizability is limited, this case highlights heart transplantation as a potential option for end-stage cardiomyopathy in LCHAD-deficient patients. With expanded newborn screening and contemporary dietary management, more individuals with LCHAD deficiency survive into adolescence and adulthood [49,58,59,60,61]. In a retrospective cohort of 16 adolescents and young adults (aged 13–31 years), cardiac involvement was documented in 9/16; three patients died suddenly, and one died of progressive dilated cardiomyopathy [58]. These findings indicate that adult-focused nutritional strategies should address not only hypoketotic hypoglycemia and exertional rhabdomyolysis, but also arrhythmic risk and cardiomyopathy [44].

## 5. Nutritional Strategies to Bypass the Enzymatic Block in LCHAD Deficiency

Food products that typically contribute most of the fat to the average human diet can be grouped into the following categories: cooking oils and spreads; fatty and processed meats; high-fat dairy; snacks and baked goods; nuts and nut butter; and fatty fish [62]. These foods supply triglycerides composed of a variety of saturated and unsaturated fatty acids [63,64], but the fatty acid chains are predominantly long (Table 4).

Thus, fats in the human diet are largely long-chain triglycerides (LCTs), i.e., molecules of glycerol esterified to three LCFAs (≥C14). Mitochondrial β-oxidation of these fatty acids supplies more than half of the energy used by high-demand tissues such as cardiac muscle, skeletal muscle, and liver. To meet the energy needs of tissues that rely on fatty acid oxidation in LCHAD deficiency, dietary therapy must bypass the enzymatic block—the loss of the long-chain 3-hydroxyacyl-CoA oxidizing activity within the mitochondrial trifunctional protein—caused by pathogenic variants in the *HADHA* gene.

### 5.1. Dietary Restriction of Long-Chain Triglycerides

In LCHAD deficiency, dietary intake of triglycerides containing fatty acids with chain lengths of 14 carbons or more can precipitate long-chain lipid toxicity, i.e., the cellular damage caused by the buildup of unoxidized long-chain 3-hydroxyacyl-derivatives [46]. Nutritional therapy should therefore restrict food products rich in LCFAs, limit daily LCT intake to no more than 10% of total energy, and ensure essential fatty-acid adequacy [55,65,66].

### 5.2. Replacing Long-Chain Triglycerides with Medium-Chain Triglycerides

Medium-chain triglycerides (MCTs)—glycerol esters of medium-chain fatty acids (MCFAs)—occur in coconut and palm kernel oils, milk and dairy products, and specialized medical foods such as purified MCT oil (Table 5).

During digestion, MCTs are hydrolyzed by lipases into glycerol and MCFAs. Unlike LCFAs, most MCFAs remain nonesterified after digestion—they are absorbed across the small intestinal mucosa and are carried via the portal vein directly to the liver [68]. Dodecanoic acid (C12) can partly follow LCFA handling, with some chylomicron incorporation and lymphatic transport; by contrast, octanoic (C8) and decanoic (C10) acids are transported predominantly through the portal circulation [68]. In the liver, MCFAs are oxidized more efficiently than LCFAs because their mitochondrial entry is largely carnitine shuttle-independent [69]. MCFAs are processed by the second β-oxidation system—an alternate set of mitochondrial enzymes with catalytic specificity for C4–C16 fatty-acyl chains (Table 1). Because this pathway remains intact, it generates acetyl-CoA to supply the tricarboxylic acid (TCA) cycle and produces NADH and FADH_2_, which pass electrons to the electron transport chain, ultimately driving cellular ATP synthesis.

Although dietary MCFAs undergo extensive oxidation on first pass through the liver, measurable amounts still reach the systemic circulation and are taken up by peripheral tissues [70]. Human cells can elongate MCFAs by two carbons before oxidizing them. In cultured skin fibroblasts from healthy individuals, octanoic (C8), decanoic (C10), and dodecanoic (C12) acids were elongated to C10, C12, and C14 intermediates, respectively [71]. The same occurred, albeit to a lesser extent, in fibroblasts from patients with LCHAD deficiency [71]. Consequently, when LCHAD-deficient patients consume MCTs containing C8, C10, and C12 MCFAs, the intracellular MCFA pool may include C8, C10, C12, and C14 species, all within the preferred substrate range of MCAD (Table 1). A small fraction of newly formed C14 may enter the long-chain β-oxidation system (Table 1). In LCHAD deficiency, such C14 flux can contribute to long-chain 3-hydroxyacylcarnitine accumulation—potentially more pronounced with C12-containing MCTs or very high MCFA intakes—which may help explain persistent circulating long-chain 3-hydroxyacylcarnitines despite low-LCFA diets. However, elongation is a minor fate of dietary MCT-derived fatty acids—less than 1% of MCFAs are elongated to LCFAs [72]. Accordingly, MCT therapy does not substantially increase the LCFA burden [72]. To minimize the potential for elongation, C8/C10-focused MCT formulations are preferred, with minimal C12 content. Monitoring long-chain 3-hydroxyacylcarnitines is advisable. Overall, the clinical impact of C14 generation via MCFA elongation appears small under standard LCHAD deficiency management.

In contrast to the liver, carnitine availability in the heart and skeletal muscle may limit mitochondrial β-oxidation of MCFAs [69]; therefore, patients with LCHAD deficiency should have plasma free carnitine and acylcarnitine levels monitored, and supplementation considered if free carnitine is low [18].

Treatment recommendations for newborns with LCHAD deficiency emphasize the use of a special MCT-containing infant formula that is low in LCTs, high in MCTs, and meets all nutritional requirements; in many guidelines, this is considered mandatory [65,66]. Because essential LCFAs are limited in such formulas, supplementation with linoleic acid, α-linolenic acid, and docosahexaenoic acid (DHA) is required [65,66]. With the introduction of solid foods, MCTs should provide 20–25% of daily caloric intake (DCI) [66].

Genotype-specific nutrition guidelines for LCHAD deficiency have not been established. However, because patients homozygous for the *HADHA* c.1528G>C variant are at higher risk of pigmentary chorioretinopathy, their nutrition plans should include DHA supplementation as a standard component; DHA plays a key role in retinal function. Although some reports describe stabilization of retinal function with optimized therapy, definitive evidence that DHA prevents or slows progression remains limited; dosing and monitoring should therefore follow specialist protocols [54,66].

Common, dose-related adverse effects of dietary MCTs/MCT oil are gastrointestinal—abdominal cramps, bloating, nausea, vomiting, and diarrhea—especially with large bolus doses or rapid titration. Tolerability improves with gradual dose escalation, taking MCT oil with meals (or thoroughly mixing it into food), and dividing the daily amount into 3–4 or more doses. In addition, MCTs are ketogenic, and blood ketone levels rise in a dose-dependent manner.

MCT oil should not be co-administered with orlistat or cetilistat. These pancreatic lipase inhibitors block intestinal triglyceride hydrolysis, thereby reducing absorption of triglyceride-based formulations.

### 5.3. Triheptanoin

**Figure 1:**
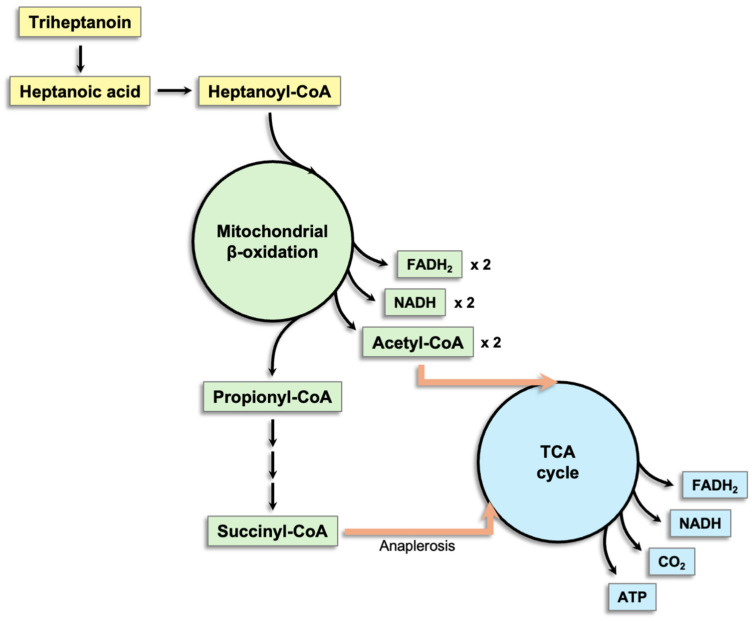
Metabolic fate of heptanoic acid derived from triheptanoin: acetyl-CoA production and succinyl-CoA–mediated anaplerosis. TCA, tricarboxylic acid.

Despite the long-standing use of MCTs to manage LCHAD deficiency, patients may still develop symptoms because conventional even-chain MCTs do not replenish TCA cycle intermediates [73]. Triheptanoin, a triglyceride composed of three odd-chain fatty acids (heptanoate, C7) esterified to glycerol, compensates for this anaplerotic deficit [74]. After intestinal hydrolysis, triheptanoin releases heptanoate, which undergoes mitochondrial β-oxidation to yield two acetyl-CoA and one propionyl-CoA per fatty acid chain. Propionyl-CoA is then converted to succinyl-CoA, providing anaplerotic input to the TCA cycle (Figure 1) [74,75]. In patients with LCHAD deficiency, in whom LCFA β-oxidation is impaired, triheptanoin can supply both energy and anaplerotic flux, thereby stabilizing mitochondrial oxidative metabolism and bioenergetics and supporting hepatic gluconeogenesis [76]. Consistent with this mechanism, several studies report that triheptanoin treatment has been associated with fewer major clinical events (e.g., rhabdomyolysis and hypoglycemia), lower hospitalization rates, and improvements in selected cardiac and hepatic outcomes [60,73,77,78,79,80,81,82].

In the liver, heptanoate is also metabolized to five-carbon (C5) ketone bodies, β-hydroxypentanoate and β-ketopentanoate, which are exported to peripheral tissues and further oxidized to yield acetyl-CoA and propionyl-CoA [74]. Accordingly, triheptanoin can increase ketonemia, particularly C5 ketones [74,78]. However, standard LCHAD deficiency dietary therapy aims to prevent fasting and ensure adequate carbohydrate intake, which limits excessive ketone production. In line with this, ketosis has not been reported in clinical studies [78], likely reflecting triheptanoin’s anaplerotic effect and its usual co-administration with low-fat, non-ketogenic meals.

Triheptanoin can be introduced after discontinuing other MCT products [83,84]. In patients transitioning from another MCT, the initial triheptanoin dose generally matches the last tolerated daily MCT amount, followed by stepwise titration by about 5% DCI every 2–3 days to a maximum of 35% DCI [83,84]. For individuals not currently taking an MCT product, a proposed starting dose is approximately 10% DCI, increased gradually over 2–3 weeks to a target of up to 35% DCI, as tolerated [83,84]. The total daily amount of triheptanoin is usually divided into at least four doses [82,83].

The potential adverse effects of triheptanoin are predominantly gastrointestinal—most commonly reported are abdominal pain, diarrhea, vomiting, and nausea [83,84]. When these symptoms occur, the daily dose may be divided into smaller, more frequent portions or temporarily reduced until they resolve. If the 35% DCI target cannot be achieved for tolerability reasons, the dose should be maintained at the patient’s maximum tolerated level. Triheptanoin is a clear, neutral-tasting oil that could be easily mixed with soft foods or drinks to improve gastrointestinal tolerability [84].

Because the digestion of triheptanoin depends on pancreatic lipase, concomitant use of pancreatic lipase inhibitors (e.g., orlistat, cetilistat) may reduce its intestinal absorption and should be avoided. Impaired intestinal absorption of triheptanoin may also occur in patients with pancreatic insufficiency; in such cases, where clinically appropriate, co-administration of pancreatic enzyme replacement therapy (PERT) with each dose may be considered to improve absorption [83].

There is limited data on the use of triheptanoin in pregnancy. Although animal studies have not demonstrated reproductive toxicity attributable to triheptanoin or its metabolites, use during pregnancy should be considered only when the potential benefits to the patient outweigh the potential risks. No data are available on excretion of triheptanoin or its metabolites into human or animal milk, on effects on lactation, or on outcomes in breastfed infants; therefore, a risk to breastfed infants cannot be excluded [83,84].

### 5.4. Carbohydrates

Current management of LCHAD deficiency emphasizes strict avoidance of fasting and regular carbohydrate-containing meals [2,55]. This approach stabilizes energy metabolism in two ways—it prevents hypoglycemia and, by shifting substrate use toward carbohydrate oxidation, partially compensates for impaired mitochondrial β-oxidation of LCFAs. In addition, carbohydrate intake activates insulin signaling, which inhibits lipolysis in adipose tissue [85,86]. The resulting reduction in LCFA release from adipocytes decreases hepatic and muscle LCFA oxidation, and lowers the formation of potentially toxic long-chain acylcarnitines [58,87].

Table 6 summarizes dietary strategies for complications of LCHAD deficiency.

### 5.5. Treatment Response Monitoring

Effective management of LCHAD deficiency should lead to at least partial reversal of clinical manifestations. The acylcarnitine profile should improve, with a fall in disease-specific long-chain 3-hydroxyacylcarnitines toward the reference range [93]. A hepatic response is reflected by normalization of transaminases (ALT, AST); testing should also include GGT, bilirubin, and albumin as an indicator of synthetic function [66]. For muscle and cardiac monitoring, plasma creatine kinase (CK) should decline and, ideally, normalize. Metabolic monitoring should also include free and total carnitine, glucose, and β-hydroxybutyrate. Routine evaluation should include ECG with Holter monitoring for arrhythmias, echocardiography, tracking of rhabdomyolysis episodes, electroretinography, liver imaging, and assessment of peripheral nerves.

## 6. Nutrigenomic Signals Elicited by Dietary Treatment in LCHAD Deficiency

### 6.1. MCT-Derived Fatty Acids Are Ligands for Nuclear Receptors

Both MCT oil—used in the treatment of long-chain fatty acid oxidation disorders—and coconut and palm kernel oils are biologically active; they deliver MCFAs that can act as ligands for peroxisome proliferator-activated receptors (PPARs). PPARs are ligand-activated transcription factors in the nuclear receptor superfamily that function as intracellular fatty acid sensors [94]. All PPAR isoforms—PPARα, PPARγ, and PPARδ—are involved in the regulation of energy homeostasis, with prominent effects on lipid metabolism, encompassing fatty acid uptake and oxidation, lipogenesis, and ketogenesis [95,96]. Octanoic (C8) and decanoic (C10) acids—the principal constituents of MCT oil—and dodecanoic acid (C12), a major component of coconut and palm kernel oils, bind PPARα, PPARγ, and PPARδ and modulate their activity [97,98]. Although MCFAs generally bind PPARs with slightly lower affinity than their long-chain counterparts, they still promote coactivator recruitment and corepressor release, converting PPARs from transcriptional repressors to activators and increasing PPAR-dependent transcription [97].

### 6.2. MCFA Effects on PPARs and Their Target Genes Are Cell- and Tissue-Specific

In 3T3-L1 adipocytes, elevated intracellular decanoic acid upregulated *Ppargc1a*, the PPAR-responsive gene encoding peroxisome proliferator-activated receptor γ coactivator-1α (PGC-1α) [97]. This effect was paralleled in vivo in the same study, with increased *Ppargc1a* expression in mouse white adipose tissue after dietary decanoic acid in triglyceride form [97]. PGC-1α coordinates metabolic reprogramming in response to nutrient availability, promotes mitochondrial biogenesis, and stimulates the expression of genes involved in fatty acid β-oxidation [99]. Consistent with these actions, decanoic acid—likely via activation of the PPAR–PGC-1α axis—attenuated lipid accumulation in adipocytes [97,98]. Extending the findings in vivo, a diet enriched in decanoic acid, provided as glyceryl tridecanoate, reduced serum TG and cholesterol concentrations—thus improving the lipid profile—lowered fasting glycemia, and enhanced insulin sensitivity in an animal model of type 2 diabetes and obesity [97].

**Figure 2:**
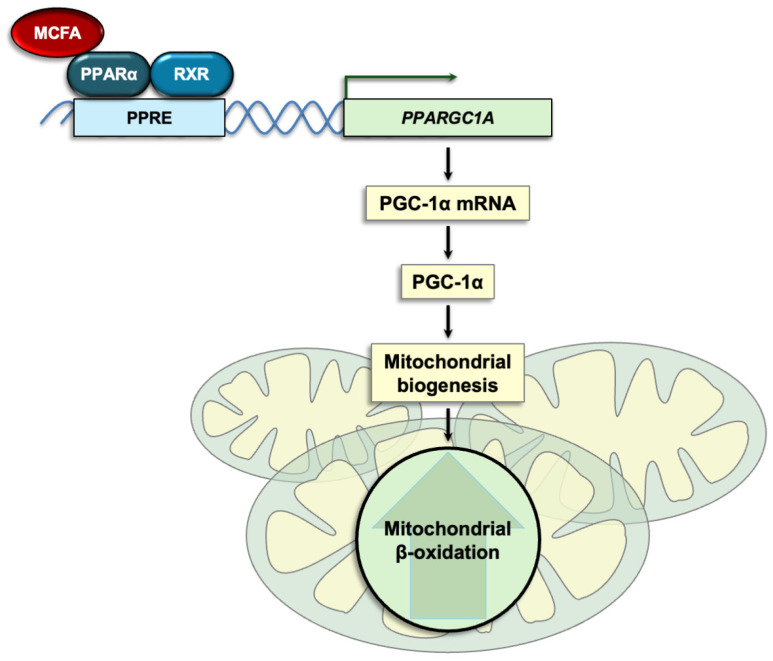
Dietary MCTs provide medium-chain fatty acids that have the potential to activate PPARs and increase PGC-1α expression, leading to enhanced mitochondrial biogenesis and induction of β-oxidation genes that remain functional, thereby increasing residual β-oxidation capacity. MCFA, medium-chain fatty acid; MCTs, medium-chain triglycerides; PGC-1α, peroxisome proliferator-activated receptor γ coactivator-1α; *PPARGC1A*, gene encoding PGC-1α; PPAR, peroxisome proliferator-activated receptor; PPRE, PPAR response element; RXR, retinoid X receptor.

By contrast, in skeletal muscle cells, the shorter-chain MCFA octanoic acid induced PGC-1α expression, whereas decanoic and dodecanoic acids did not [100]. Myotubes exposed to octanoic acid exhibited increased mitochondrial volume, higher expression of genes encoding key mediators of mitochondrial fission and mitophagy, and enhanced oxidative phosphorylation [100]. Octanoic acid also promoted proliferation, differentiation, and maturation of these cells [100]. These findings suggest that octanoic acid induces PGC-1α-driven mitochondrial biogenesis and improves mitochondrial quality control in skeletal muscle cells (Figure 2). A study in mice reported similar findings, showing that a diet supplemented with octanoic acid—in the form of caprylic triglyceride—upregulated PGC-1α in skeletal muscle, induced mitochondrial biogenesis, and shifted skeletal muscle toward a more oxidative phenotype by increasing the abundance of mitochondria-rich fibers [101]. Consistent with improved muscle oxidative capacity, an octanoic acid-enriched diet increased skeletal muscle fibers’ resistance to fatigue [101].

In human liver cells, octanoic and decanoic acids comparably induced *CPT1A*, an established PPARα-responsive gene that encodes carnitine palmitoyltransferase 1A (CPT1A), which controls the mitochondrial uptake of fatty acids [102,103]. These findings support PPARα transcriptional activation in the liver by both MCFAs. In HepG2 hepatocytes, the same MCFAs similarly activated PPARγ [98]. Conversely, the LCFAs palmitic acid (C16) and oleic acid (C18:1) reduced *CPT1A* expression [102,103]. In rats, dietary MCT oil—composed mainly of TGs with octanoic and decanoic acids—activated PPARα and upregulated hepatic expression of acyl-CoA oxidase (ACO), a canonical PPARα target that catalyzes the first, rate-limiting step of peroxisomal β-oxidation of fatty acids [104]. Accompanying increases in mitochondrial respiratory chain complexes III and V [104] suggest that dietary MCTs may enhance hepatic fatty acid oxidation and mitochondrial respiratory capacity. Octanoic and decanoic acids activate PPARα-driven transcription in the liver; whether MCFAs also upregulate PPAR expression per se remains uncertain. An in vivo study in mice showed that a decanoate-enriched diet raised hepatic PPARα and PPARγ expression [97]. In vitro, by contrast, MCFAs did not change PPARα expression in human hepatocyte lines [102,103]. MCFAs may further initiate hepatic PPAR-dependent transcription indirectly—for example, through cooperating factors.

To our knowledge, no study has directly quantified PPAR activation or PPAR-target gene responses attributable to dietary MCTs or individual MCFAs in patients with isolated LCHAD deficiency. Because LCHAD deficiency is very rare, investigations of MCFA-evoked molecular mechanisms in affected patients are likewise scarce. Existing evidence for PPAR engagement comes from patients with mitochondrial trifunctional protein (MTP/TFP) deficiency, in whom fatty acid-driven endogenous PPARα activation was demonstrated in skin fibroblasts [105]. These data suggest that nutrigenomic mechanisms elicited by MCT-derived fatty acids and involving PPARs—shown in vitro and in animal models—are likely active in patients with LCHAD deficiency, although this remains to be confirmed.

Given the tissue-specific responses to MCFAs, a targeted approach to MCT therapy in LCHAD-deficient patients is warranted. Although no LCHAD deficiency-specific clinical trial has established an optimal C8:C10 ratio, MCT composition should be guided by the patient’s clinical complications (Table 6) and tolerability. If muscle manifestations—rhabdomyolysis and cardiomyopathy—are the primary concern, a C8-dominant formulation—given its faster oxidation, rapid mitochondrial uptake, and favorable signaling in muscle models—should be used, with the ratio and dose adjusted for gastrointestinal tolerance. When hepatic disease predominates, a C8-rich or mixed C8/C10 formulation is reasonable, as some patients with LCHAD deficiency tolerate mixed oils better.

### 6.3. MCFAs Increase FGF21 and Activate AMPK

After ingestion, dietary MCFAs first reach the liver, where they not only are oxidized but also modulate hepatic gene expression [106]. They are potent natural inducers of fibroblast growth factor 21 (FGF21) [106], a predominantly liver-derived endocrine hormone that regulates systemic lipid and glucose homeostasis, stimulates glucose uptake in adipose tissue and skeletal muscle, and increases insulin sensitivity [107,108,109]. In mice, dietary MCFAs provided as triglycerides of octanoic (C8) and decanoic (C10) acids upregulated hepatic *Fgf21* and increased circulating FGF21 [106]. By contrast, LCFAs did not affect *Fgf21* expression or plasma FGF21 [106]. The *FGF21* gene is a direct target of PPARα in both mouse and human [107,108]. MCFAs can act as PPAR agonists [97,98], promoting hepatic FGF21 production. In the liver, *FGF21* transcription is also driven synergistically by the endoplasmic reticulum membrane-anchored transcription factor cAMP-responsive element-binding protein, hepatocyte-specific (CREBH), and the nuclear receptor PPARα [110]. In mice fed C8–C10 MCFAs, hepatic levels of the cleaved (active) form of CREBH increased [106]. In the same study, animals on an MCFA-enriched diet—compared with an LCFA-enriched diet—had lower liver TG content and reduced plasma TGs [106]. Recent genomic data indicate that FGF21 stimulates hepatic fatty acid β-oxidation, suppresses lipogenesis, and protects the liver from lipid overload in humans and rodents [107]. Consistent with these effects, dietary octanoic and decanoic acids lowered hepatic lipid content in an FGF21-dependent manner [106]. In primary mouse hepatocytes, decanoic acid activated p38 kinase, with concomitant CREB phosphorylation and induction of gluconeogenic genes [111]. FGF21 also upregulates hepatic PGC-1α expression and increases mitochondrial respiration and oxidative phosphorylation [107].

Additionally, FGF21 can modulate energy expenditure by stimulating AMP-activated protein kinase (AMPK), an intracellular energy sensor that regulates mitochondrial function [112]. In vitro, FGF21 activated AMPK in human and murine adipocytes; in vivo evidence also supported increased AMPK activity [109]. Because active AMPK induces and directly phosphorylates PGC-1α [113], AMPK activation can trigger the mitochondrial biogenesis program. In mice, AMPK activation contributed to MCT feeding-induced upregulation of PGC-1α and increased expression of mitochondrial respiratory chain components in skeletal muscle [101].

Human data specific to LCHAD deficiency and FGF21 are scarce, and adult data are lacking. In a multicenter pediatric study, diet-treated children with LCHAD deficiency had low-normal serum FGF21, whereas one untreated infant in metabolic crisis showed a marked elevation [114]. Low-normal values can reflect good metabolic control—i.e., absence of active hepatic lipid stress and catabolic stress—but they may also simply indicate sampling outside an inducer window (e.g., not after prolonged fasting, carbohydrate overfeeding, or protein restriction). The effects of dietary MCTs on FGF21 in LCHAD deficiency merit investigation, particularly because FGF21, a stress-responsive hepatokine, was recently shown to lower hepatic TGs, reduce hepatic cholesterol, and reverse fibrosis via coordinated actions on the CNS and liver, thereby improving metabolic health [115].

## 7. Final Remarks

Identification of pathogenic *HADHA* variants in an affected proband enables cascade carrier testing for at-risk relatives and permits prenatal and preimplantation genetic testing. In parallel, there is a critical need to develop cost-effective gene-targeted therapies, a priority for future research and, ultimately, clinical translation. More broadly, better disease-modifying strategies are urgently needed. At present, care for LCHAD deficiency relies on strict fasting avoidance and a fat-restricted diet supplemented with MCTs or odd-chain triglycerides. Because low-fat diets can predispose to fat-soluble vitamin deficiencies, careful monitoring and targeted supplementation are recommended.

Odd-chain triglycerides replenish TCA cycle intermediates, stabilizing TCA flux and oxidative phosphorylation. This support can improve metabolic resilience in tissues with high energy demand and during fasting, exercise, or intercurrent illness. MCTs yield MCFAs that are absorbed more rapidly than LCFAs. Dietary MCFAs can help maintain glycemic control and support ketogenesis, increasing the availability of oxidizable energy substrates. Nutrients emphasized in dietary therapy for LCHAD deficiency and their metabolites can also act as molecular signals that modulate gene expression, altering transcriptional programs involved in mitochondrial energy metabolism.

Personalized nutrition based on these principles often improves symptoms and quality of life. However, long-term prospective studies are still needed to determine whether lowering circulating long-chain 3-hydroxyacylcarnitines through dietary interventions reduces the incidence or slows the progression of LCHAD deficiency-related complications.

## Figures and Tables

**Table 1:** β-oxidation systems in human mitochondria.

Enzyme/Activity	Gene	Substrates	Activity
**Very-Long- and Long-Chain β-oxidation System**			
VLCAD	*ACADVL*	C12–C24	very-long-chain acyl-CoA dehydrogenase, EC:1.3.8.9
ACAD9	*ACAD9*	–	moonlighting protein, probably oxidating long-chain acyl-CoAs
LCAD	*ACADL*	C8–C18	long-chain acyl-CoA dehydrogenase, EC:1.3.8.8
MTP-LCEH	*HADHA*	C8–C24	long-chain enoyl-CoA hydratase, EC:4.2.1.17
MTP-MLCLAT	*HADHA*	C18	monolysocardiolipin acyltransferase, EC:2.3.1
MTP-LCHAD	*HADHA*	C6–C24	long-chain 3-hydroxyacyl-CoA dehydrogenase, EC:1.1.1.211
MTP-LCKAT	*HADHB*	C6–C24	long-chain 3-ketoacyl-CoA thiolase, EC:2.3.1.155/EC:2.3.1.16
**Medium- and Short-Chain β-oxidation System**			
MCAD	*ACADM*	C6–C16	medium-chain acyl-CoA dehydrogenase, EC:1.3.8.7
SCAD	*ACADS*	C4–C6	short-chain acyl-CoA dehydrogenase, EC:1.3.8.1
ECHS1	*ECHS1*	C4–C16	enoyl-CoA hydratase, EC:4.2.1.17/EC:5.3.3.8
HADH	*HADH*	C4–C16	hydroxyacyl-CoA dehydrogenase, EC:1.1.1.35
KAT	*ACAA2*	C4–C16	3-ketoacyl-CoA thiolase, EC:2.3.1.16

Table created by the authors from data in [3,5,9,10,11,12,13,14].

**Table 4:** Common food products that supply the majority of dietary fat in the average human diet.

Source	Fatty Acids					
	<C14	C14	C16	C18	C20	>C20
	**Cooking Oils and Spreads [per 100 g] (% total fat)**					
Canola oil	0 g	0 g	4.51 g (4.55%)	92.33 g (93.1%)	1.32 g (1.33%)	0.33 g (0.33%)
Corn oil	0 g	0.03 g (0.03%)	10.9 g (11.6%)	82.8 g (87.5%)	0.25 g (0.26%)	0.21 g (0.22%)
Olive oil	0 g	0 g	12.6 g (13.0%)	83.8 g (86.2%)	0.31 g (0.32%)	0.13 g (0.13%)
Soybean oil	0 g	0.10 g (0.10%)	9.82 g (10.2%)	85.1 g (88.6%)	0.19 g (0.20%)	0.48 g (0.50%)
Sunflower oil	0.01 g	0.05 g (0.05%)	4.60 g (4.94%)	86.8 g (93.2%)	0.26 g (0.28%)	1.15 g (1.23%)
Butter	11.6 g (14.4%)	7.44 g (9.29%)	23.5 g (29.4%)	37.0 g (46.2%)	0.10 g (0.12%)	0 g
Lard	0.30 g (0.32%)	4.00 g (4.21%)	65.0 g (68.4%)	25.7 g (27.1%)	0 g	0 g
	**Fatty and Processed Meats [per 100 g] (% total fat)**					
Pork, shoulder	0.03 g (0.19%)	0.22 g (1.38%)	4.39 g (27.5%)	11.1 g (69.5%)	0.23 g (1.45%)	0 g
Salami, pork	0 g	0.52 g (1.66%)	8.86 g (28.3%)	21.8 g (69.6%)	0.16 g (0.51%)	0 g
	**High-Fat Dairy [per 100 g] (% total fat)**					
Cheese, Gouda	4.19 g (16.8%)	3.04 g (12.2%)	7.74 g (31.0%)	9.97 g (40.0%)	0 g	0 g
Cheese, cream	3.54 g (11.2%)	3.63 g (11.5%)	10.1 g (31.7%)	14.1 g (44.4%)	0.17 g (0.52%)	0.05 g (0.16%)
	**Snacks and Baked Goods [per 100 g] (% total fat)**					
Muffins, blueberry	0 g	0 g	1.91 g (12.0%)	13.8 g (86.3%)	0.13 g (0.80%)	0.08 g (0.50%)
Snacks, potato sticks	0 g	0.27 g (0.82%)	8.08 g (24.6%)	24.6 g (74.7%)	0.03 g (0.09%)	0 g
	**Nuts and Nut Butter [per 100 g] (% total fat)**					
Walnuts	0 g	0 g	4.40 g (7.07%)	57.6 g (92.6%)	0.13 g (0.22%)	0 g
Peanuts	0 g	0.03 g (0.05%)	5.16 g (11.13%)	40.50 g (87.39%)	0.66 g (1.43%)	0 g
Peanut butter	0.02 g (0.05%)	0.05 g (0.11%)	5.50 g (12.1%)	39.3 g (86.2%)	0.81 g (1.76%)	0 g
	**Fatty Fish [per 100 g] (% total fat)**					
Herring	0.02 g (0.20%)	0.55 g (6.59%)	1.97 g (23.5%)	1.86 g (22.2%)	1.49 g (17.7%)	1.76 g (20.9%)
Mackerel	0.02 g (0.14%)	0.67 g (5.67%)	2.85 g (24.0%)	3.08 g (25.9%)	2.12 g (17.9%)	3.02 g (25.4%)
Salmon	0 g	0.56 g (5.33%)	2.67 g (25.6%)	4.28 g (41.0%)	1.22 g (11.7%)	1.50 g (14.4%)

C14, saturated and unsaturated fatty acids with chain lengths of 14 carbons; C16, saturated and unsaturated fatty acids with chain lengths of 16 carbons; C18, saturated and unsaturated fatty acids with chain lengths of 18 carbons; C20, saturated and unsaturated fatty acids with chain lengths of 20 carbons. Table created by the authors from data in [62].

**Table 5:** Dietary sources of medium-chain fatty acids.

Source	Caproic (Hexanoic) Acid C6	Caprylic (Octanoic) Acid C8	Capric (Decanoic) Acid C10	Lauric (Dodecanoic) Acid C12	Other Fatty Acids >C12
	**Natural Dietary Sources [per 100 g] (% total fat)**				
Coconut oil	0.477 g (0.53%)	6.80 g (7.51%)	5.39 g (5.95%)	41.8 g (46.2%)	35.9 g (39.7%)
Palm kernel oil	0.2 g (0.21%)	3.3 g (3.49%)	3.7 g (3.92%)	47.0 g (49.7%)	40.3 g (42.7%)
Milk, cow, whole	0.054 g (1.97%)	0.034 g (1.24%)	0.084 g (3.07%)	0.097 g (3.54%)	2.40 g (87.6%)
Milk, goat	0.09 g (2.65%)	0.10 g (2.71%)	0.26 g (7.33%)	0.12 g (3.50%)	2.89 g (81.3%)
Milk, sheep	0.15 g (2.35%)	0.14 g (2.24%)	0.40 g (6.48%)	0.24 g (3.87%)	5.18 g (83.8%)
Milk, human	0 g	0 g	0.063 g (1.59%)	0.256 g (6.48%)	3.63 g (93.2%)
	**Medical Foods [per 100 mL] (% total fat)**				
MCT oil Nutricia (coconut and/or palm oil)	0.44 g (0.50%)	52.1 g (59.6%)	34.6 g (39.6%)	0.17 g (0.19%)	0.09 g (0.10%)

MCT, medium-chain triglyceride. Table created by the authors from data in [62,67].

**Table 6:** Clinical manifestations, pathophysiology, and dietary management in LCHAD deficiency.

Clinical Manifestations	Pathophysiology	Dietary Strategies
Hypoketotic hypoglycemia	Impaired LCFA β-oxidation Hepatic energy deficit Suppression of gluconeogenesis Accumulation of long-chain 3-hydroxyacyl intermediates	Frequent carbohydrate feeding Control carbohydrate intake during decompensation Restrict LCTs and add MCTs or triheptanoin Consider overnight feeds in infants Supplement essential fatty acids
Rhabdomyolysis (skeletal muscle myopathy)	Muscle energy failure Mitochondrial stress Toxic acylcarnitine accumulation	Pre-exercise carbohydrate Personalized MCTs or triheptanoin Reduction in LCTs Maintain hydration and temperature Avoid excessive physical activity Supplement carnitine, if deficient (monitor free vs. acylcarnitine)
Cardiomyopathy and arrhythmias	Impaired LCFA β-oxidation Energy deficit due to myocardial preference for LCFAs as an energy substrate Accumulation of arrhythmogenic long-chain acylcarnitines	Restrict LCTs and add MCTs or triheptanoin Ensure essential fatty acids sufficiency Consider DHA supplementation Avoid fasting
Hepatic dysfunction	Impaired LCFA β-oxidation Microvesicular steatosis Oxidative stress	Frequent carbohydrate feeding Restrict LCTs and add MCTs or triheptanoin Avoid prolonged fasting (long medical procedures) Supplement fat-soluble vitamins and essential fatty acids
Pigmentary retinopathy	Altered retinal DHA metabolism Mitochondrial dysfunction Chronic metabolite exposure	Supplement DHA and essential fatty acids Avoid decompensations Restrict LCTs and add MCTs
Peripheral neuropathy	Chronic energy deficit Mitochondrial injury in peripheral nerves	Avoid fasting Illness carbohydrate regimen Restrict LCTs and add MCTs or triheptanoin Supplement DHA and essential fatty acids

DHA, docosahexaenoic acid; LCFA, long-chain fatty acid; LCTs, long-chain triglycerides; MCTs, medium-chain triglycerides. Table created by the authors from data in [2,18,20,37,44,51,55,65,88,89,90,91,92].

## Data Availability

No new data were created or analyzed in this study. Data sharing is not applicable to this article.
